# Therapeutic Potential of *Gynostemma pentaphyllum* (Thunb.) Makino Against COVID-19 Identified Through Network Pharmacology

**DOI:** 10.3390/ph18121851

**Published:** 2025-12-04

**Authors:** Min Ho Kim, Jin Ah Won, Jun Sang Yu, Su Min Kim, Dong Keun Lee, Xiang-Lan Piao, Hye Hyun Yoo

**Affiliations:** 1 Pharmacomicrobiomics Research Center, College of Pharmacy, Hanyang University, Ansan 15588, Republic of Korea; mhk2066@hanyang.ac.kr (M.H.K.); jennywon97@hanyang.ac.kr (J.A.W.); yujs0804@jejunu.ac.kr (J.S.Y.); ksm990201@naver.com (S.M.K.); 2 New Drug Development Center, Daegu-Gyeongbuk Medical Innovation Foundation, Daegu 41061, Republic of Korea; 3 College of Pharmacy, Jeju National University, Jeju 63243, Republic of Korea; 4 GENECT, Konkuk University Campus Town, Seoul 05073, Republic of Korea; pydk42@gmail.com; 5 Key Laboratory of Ethnomedicine, Ministry of Education, Minzu University of China, Beijing 100081, China

**Keywords:** *Gynostemma pentaphyllum*, flavonoids, triterpenoid saponins, network pharmacology, molecular docking, COVID-19

## Abstract

**Background/Objectives:** The ongoing challenges posed by COVID-19 have highlighted the need for multi-target therapeutic strategies addressing both acute immune responses and systemic complications. *Gynostemma pentaphyllum* (Thunb.) Makino, a traditional herbal medicine rich in flavonoids and saponins, exhibits diverse pharmacological activities, including immunomodulatory and cardiovascular effects. In this study, we investigated the potential of *G. pentaphyllum* as a complementary treatment for COVID-19 using a network pharmacology approach combined with molecular docking analysis. **Methods:** To delve into the therapeutic mechanisms of *G. pentaphyllum*, we identified 59 active compounds and predicted 408 protein targets, of which 19 overlapped with COVID-19-associated genes, including *IL1B, IL6, TNF, ACE*, and *REN*. GO and KEGG enrichment analyses were conducted to determine relevant biological processes and pathways, focusing on cytokine signaling, inflammatory responses, and the renin–angiotensin system. Network analyses evaluated interactions of flavonoids and triterpenoid saponins with immunological, inflammatory, renin–angiotensin system, and host entry pathways. Molecular docking was performed to validate the binding affinities of key compounds to their predicted targets. **Results:** The compound–target–pathway network revealed class-specific patterns: flavonoids primarily mapped to immuno-inflammatory nodes, whereas triterpenoid saponins were enriched for renin–angiotensin system/host-entry–related targets. Docking energies spanned −6.1 to −11.9 kcal/mol, with six compound–target pairs ≤ −10.0 kcal/mol. Notably, NOS2–rutin (−11.9 kcal/mol), NOS2–gypenoside LI (−11.6 kcal/mol), and ACE–gypenoside LI (−11.3 kcal/mol) showed the strongest affinities. **Conclusions:** These findings provide evidence that *G. pentaphyllum* exerts therapeutic effects through the complementary actions of flavonoid and saponin components, each modulating distinct molecular pathways. This dual mechanistic potential underscores the value of *G. pentaphyllum* as a versatile therapeutic for COVID-19 therapy.

## 1. Introduction

Coronavirus disease 2019 (COVID-19), caused by the severe acute respiratory syndrome coronavirus 2 (SARS-CoV-2), continues to pose a major threat to global public health [1]. While vaccines and antiviral agents have mitigated the severity of acute infection in many regions, the emergence of viral variants and the growing incidence of post-acute sequelae—collectively referred to as long COVID—highlight the limitations of current therapeutic options [2,3]. Long COVID, characterized by persistent symptoms such as fatigue, cognitive impairment, and inflammation-related disorders, presents a new clinical challenge that requires novel and integrative treatment approaches [4]. In this context, traditional herbal medicine—long used in the management of respiratory and systemic inflammatory conditions—offers a valuable reservoir of bioactive compounds with potential antiviral, anti-inflammatory, and immunomodulatory properties [5].

*Gynostemma pentaphyllum* (Thunb.) Makino, a widely used herb in East Asian traditional medicine, has been reported to exhibit a broad spectrum of pharmacological activities, including anti-inflammatory, antioxidant, antitumor, hepatoprotective, and immunomodulatory effects [6]. These therapeutic properties are primarily attributed to two major classes of bioactive compounds: flavonoids and saponins [7]. Among these, specific compounds such as quercetin, ombuin, kaempferol, and gypenoside XVII have been shown in previous studies to inhibit pro-inflammatory cytokines, regulate immune responses, and interfere with viral replication pathways—activities that are particularly relevant to the treatment of COVID-19 [8,9,10,11]. Based on these characteristics, *G. pentaphyllum* extracts may hold potential as a complementary therapeutic agent against COVID-19. However, to date, no studies have systematically examined the efficacy or underlying mechanisms of *G. pentaphyllum* in this context.

Network pharmacology has emerged as a powerful in silico strategy for investigating the multi-component, multi-target nature of herbal medicines [12]. By integrating bioinformatics tools, chemical-target prediction, disease gene annotation, pathway enrichment analysis, and protein–protein interaction (PPI) networks, network pharmacology enables researchers to comprehensively map the complex interactions between natural compounds and disease-related targets [13]. This approach has been increasingly adopted in natural product research to uncover molecular mechanisms, identify therapeutic targets, and guide rational drug development [14].

Despite extensive pharmacological investigations into *G. pentaphyllum*, prior network-pharmacology studies have predominantly addressed non-viral indications without directly interrogating coronavirus-related processes or the renin–angiotensin system (RAS). As summarized in previous *G. pentaphyllum* network-pharmacology reports, these works mapped *G. pentaphyllum* constituents to PI3K–Akt, NF-κB, MAPK, JAK–STAT, and cancer-associated modules across liver, metabolic, inflammatory, and oncologic settings but did not evaluate COVID-19-specific immune dysregulation. To our knowledge, no in silico systems study has systematically examined *G. pentaphyllum* against COVID-19 pathophysiology using an integrated network-pharmacology-docking workflow.

In this study, we aimed to explore the therapeutic potential of *G. pentaphyllum* extract as a candidate treatment for COVID-19. To support this, we investigated the putative molecular targets and pathways associated with its bioactive compounds using a network pharmacology approach. Furthermore, molecular docking analysis was employed to assess the binding affinities between representative *G. pentaphyllum*-derived compounds and key COVID-19-related target proteins, thereby providing mechanistic insights into its possible mode of action. Through this integrative approach, we aim to provide a scientific rationale for the potential development of *G. pentaphyllum* extract as a complementary therapeutic agent for COVID-19.

## 2. Results

### 2.1. Network Pharmacology Analysis

**Figure 1:**
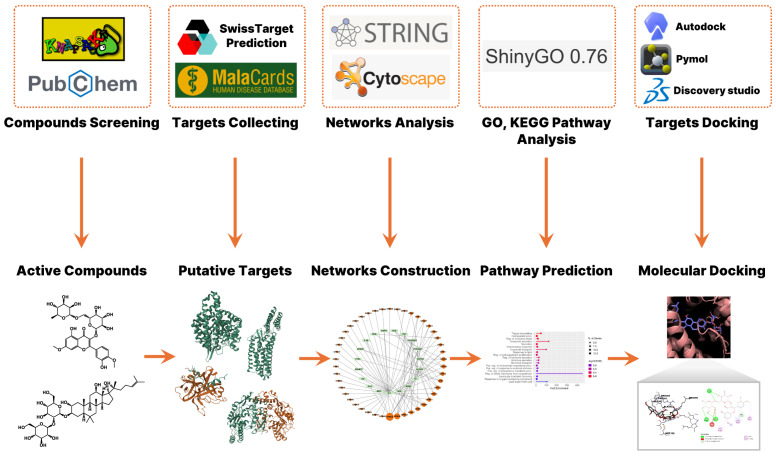
Network pharmacology for identifying mechanisms of *G. pentaphyllum* in treating COVID-19.

To investigate the potential therapeutic mechanisms of *G. pentaphyllum* in the treatment of COVID-19, we performed a network pharmacology analysis comprising compound screening, target prediction, disease-related gene identification, and network construction (Figure 1). A total of 59 active compounds were initially identified from *G. pentaphyllum* using the KNApSAcK and PubChem databases. Oral bioavailability and drug-likeness of these compounds were calculated using the SwissADME platform (Table S3). From these compounds, 408 putative protein targets were predicted. In parallel, 143 COVID-19-related targets were retrieved from the MalaCards database, which aggregates disease-related genes from multiple biomedical sources. A set of 19 overlapping targets between *G. pentaphyllum*-derived compounds and COVID-19-associated genes was identified, suggesting potential therapeutic relevance.

**Figure 2:**
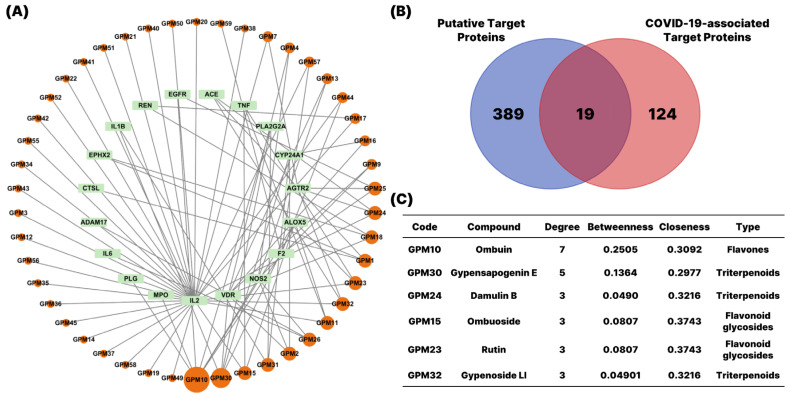
Network pharmacology-based identification of candidate targets and active components of *G. pentaphyllum* against COVID-19. (**A**) Construction of Compound-Target network. Different active compounds (orange circular nodes) are displayed around the periphery, with node size reflecting the number of predicted targets. Putative target proteins (green rectangular nodes) are positioned inside the circle defined by the active compounds. (**B**) Venn diagram showed the overlap between the putative target proteins of *G. pentaphyllum* and COVID-19-associated targets. (**C**) Major active compounds of *G. pentaphyllum* related to COVID-19.

To visualize these interactions, we constructed a compound–target (CT) network using Cytoscape 3.10.1 (Figure 2). The network consisted of two types of nodes: orange circular nodes representing active compounds, and green rectangular nodes representing their corresponding protein targets. The size of each compound node reflected the number of targets associated with that compound. Notably, three flavonoids—ombuin, ombuoside, rutin—and three triterpenoids—damulin B, gypensapogenin E, gypenoside LI—demonstrated high degrees of connectivity in the compound–target network. These compounds interacted with multiple key target proteins, suggesting their central roles in mediating the pharmacological effects of *G. pentaphyllum.*

### 2.2. Protein–Protein Interaction

**Figure 3:**
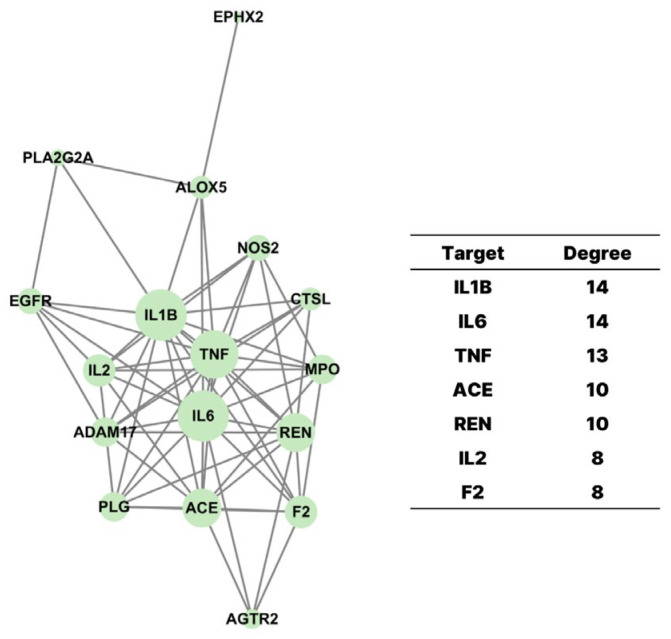
The Protein–Protein Interaction (PPI) network. The top 17 intersecting target proteins were shown in circular nodes with different sizes. The size of the nodes expressed the interaction degree on the network.

To further investigate the biological relevance of the 19 overlapping targets between *G. pentaphyllum* compounds and COVID-19-associated genes, a protein–protein interaction (PPI) network was constructed using the STRING database and visualized through Cytoscape 3.10.1 (Figure 3). The resulting network revealed several highly connected hub proteins, including IL1B, IL6, TNF, ACE, and REN, which are central regulators of inflammatory and cardiovascular pathways known to play key roles in COVID-19 pathogenesis. The size of each node represented its degree of connectivity, with IL6 and TNF exhibiting the highest degrees, highlighting their importance within the interaction network.

### 2.3. GO and KEGG Pathway Enrichment Analysis

**Figure 4:**
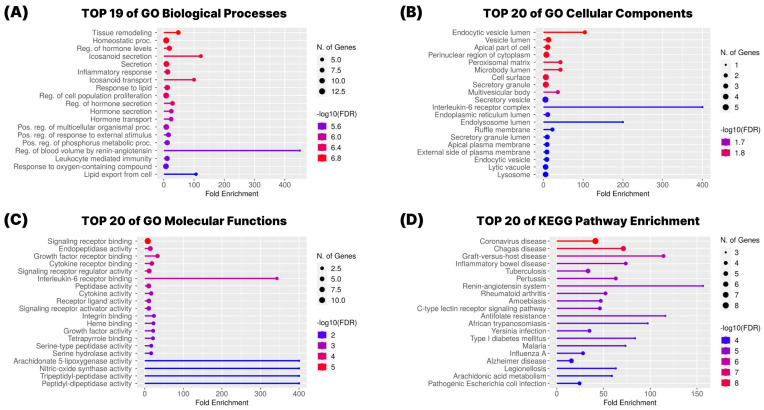
GO and KEGG Pathway Enrichment Analysis. (**A**) Biological process, (**B**) cellular components, and (**C**) molecular functions of Gene Ontology (GO) analysis. (**D**) Kyoto Encyclopedia of Genes and Genomes (KEGG) pathway enrichment analysis.

To elucidate the biological functions and signaling pathways associated with the 19 overlapping targets between *G. pentaphyllum* compounds and COVID-19-related genes, we performed Gene Ontology (GO) and Kyoto Encyclopedia of Genes and Genomes (KEGG) pathway enrichment analyses using the ShinyGO 0.77 platform (Figure 4).

The GO enrichment analysis was conducted across three functional categories: biological process (BP), cellular component (CC), and molecular function (MF). In the biological process category (Figure 4A), the targets were mainly associated with immune and inflammatory regulation, with significant enrichment in terms such as inflammatory response, regulation of cytokine production, response to lipopolysaccharide, and immune effector process. These findings indicate that *G. pentaphyllum*-related targets play crucial roles in modulating host defense mechanisms relevant to COVID-19. In the cellular component category (Figure 4B), enriched terms included external side of plasma membrane, membrane raft, and secretory granule membrane, suggesting that many of these targets are membrane-associated or secreted proteins, consistent with their involvement in cytokine signaling and immune cell interactions. Regarding molecular function (Figure 4C), the most prominent terms included cytokine activity, receptor binding, and enzyme binding, reinforcing the targets’ involvement in immune signaling pathways.

KEGG pathway enrichment analysis further revealed that the 19 targets are significantly involved in pathways central to COVID-19 pathology (Figure 4D). These included the cytokine–cytokine receptor interaction, TNF signaling pathway, IL-17 signaling pathway, NF-κB signaling pathway, and the renin–angiotensin system. These pathways are closely associated with hyperinflammation, cytokine storm, and cardiovascular complications frequently observed in severe COVID-19 cases. The enrichment of these pathways suggests that *G. pentaphyllum* may act through the modulation of both inflammatory signaling and the renin–angiotensin axis to exert potential therapeutic effects.

### 2.4. Compound–Target–Pathway (CTP) Network

**Figure 5:**
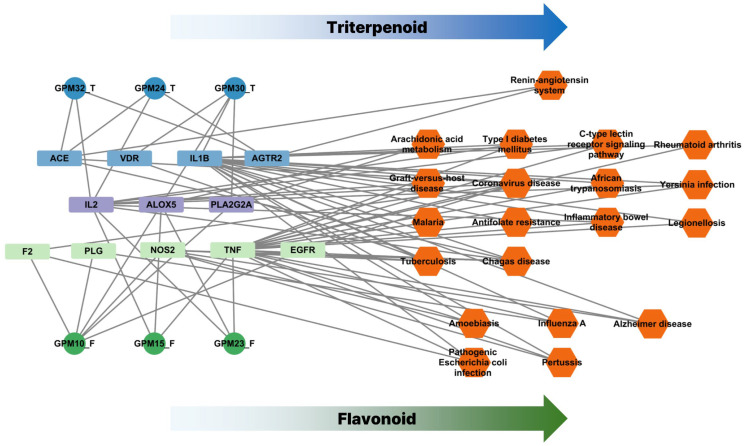
The compound-target-pathway (CTP) network. The compound–target–pathway (CTP) network constructed for *G. pentaphyllum*. Circular nodes represent active compounds, rectangular nodes represent predicted protein targets, and hexagonal nodes represent KEGG pathways. Targets predominantly linked to triterpenoid compounds are shown in blue, flavonoid-related targets in green, and shared targets in purple.

To further elucidate the mechanistic landscape of *G. pentaphyllum*, a compound–target–pathway (CTP) network was constructed, as illustrated in Figure 5. This network visually demonstrates the multi-level interactions among active compounds, their predicted protein targets, and the associated biological pathways. Notably, the bioactive compounds identified from *G. pentaphyllum* clustered into two major phytochemical classes: flavonoids and triterpenoids. These two classes showed distinct interaction profiles within the network, suggesting different modes of action. Flavonoid-type compounds (e.g., GPM10, GPM15, GPM23) were predominantly associated with inflammation-related targets such as TNF, NOS2, EGFR, and IL2, and enriched in signaling pathways related to immune modulation and infection control, including cytokine signaling, tuberculosis, and influenza pathways. In contrast, triterpenoid-type compounds (e.g., GPM24, GPM30, GPM32) interacted with a separate subset of targets including ACE, AGTR2, VDR, and were more involved in cardiovascular and renin–angiotensin system-related pathways, as well as metabolic and viral disease modules. This division of target specificity highlights a dual mechanism of action: flavonoids may primarily exert immunomodulatory and anti-inflammatory effects, while triterpenoids appear to contribute to cardiovascular regulation and viral entry interference. The presence of both compound classes in *G. pentaphyllum* underscores its potential as a broad-spectrum therapeutic candidate that targets COVID-19 pathophysiology through complementary mechanisms.

### 2.5. Molecular Docking Analysis

To validate the interaction between bioactive compounds of *G. pentaphyllum* and key protein targets related to COVID-19, molecular docking simulations were performed against 12 representative host or viral proteins. Docking results for seven major compounds are summarized in Table 1. Docking simulations revealed binding energies ranging from −6.1 to −11.9 kcal/mol, supporting that the majority of key compounds and protein targets identified through network pharmacology exhibit substantial mutual binding potential relevant to COVID-19 pathophysiology. The docking protocol was validated, as the heavy-atom root-mean-square deviation (RMSD) values between the redocked and crystallographic poses were ≤2.0 Å (or within a comparable range).

Notably, flavonoid-type compounds such as rutin (GPM23), ombuin (GPM10), and ombuoside (GPM15) generally exhibited strong affinities toward immune-related targets including NOS2, PLG, and TNF, with docking scores often exceeding −9.0 kcal/mol. On the other hand, triterpenoid-type compounds such as gypensapogenin E (GPM30), damulin B (GPM24), and gypenoside LI (GPM32) preferentially targeted host cell entry and cardiovascular-related proteins such as ACE, AGTR2, and VDR, with several docking scores below −10.0 kcal/mol.

**Figure 6:**
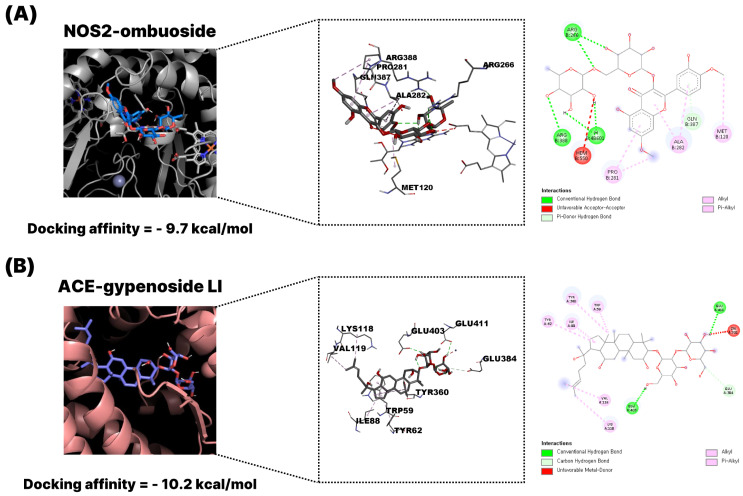
Representative molecular docking diagrams of *G. pentaphyllum* active compounds and key targets associated with COVID-19 using AutoDock Vina (version 1.1.2). (**A**) Docking model of ombuoside (GPM15, flavonoid) with NOS2. (**B**) Docking model of gypenoside LI (GPM32, triterpenoid) with ACE. The visualization includes the overall protein–ligand complex, binding site interactions (3D), and 2D interaction mapping.

To visually represent the docking interactions, representative binding models of ombuoside (GPM15, flavonoid) and gypenoside LI (GPM32, triterpenoid) were selected and illustrated. As shown in Figure 6A, ombuoside demonstrated a strong binding affinity to NOS2 (−11.0 kcal/mol), a key enzyme in pro-inflammatory signaling. The docking pose revealed multiple conventional hydrogen bonds and π–π stacking interactions with residues such as GLY202, CYS200, TYR489, and TRP194. These interactions stabilize the ligand within the active site pocket, supporting its potential role in immunomodulation and inflammation suppression. Docking and inhibition studies on diverse iNOS ligands have repeatedly shown that Cys200, Gly202, Trp194 and Tyr489 make key contacts stabilizing inhibitors in the substrate access channel and are associated with reduced NO production [15,16,17]. Thus, the engagement of these residues by ombuoside supports a plausible mechanism in which tight binding in the heme-proximal pocket hinders L-arginine oxidation and thereby attenuates NO-mediated inflammatory responses. Figure 6B presents the docking of gypenoside LI with ACE (−11.3 kcal/mol), a central component of the renin–angiotensin system and viral entry mechanism. The triterpenoid scaffold of gypenoside LI formed extensive hydrogen bonds and hydrophobic contacts with residues such as ARG124, PHE570, MET223, and PRO407, suggesting interference with ACE activity and possible inhibition of SARS-CoV-2 spike protein binding. Met223 and Phe570 belong to the S1/S2 subsites that line the substrate-binding channel around the catalytic HEXXH zinc-binding motif, whereas Arg124 and Pro407 are positioned along the neighboring substrate-access channel; residues in these regions are known to govern the binding and potency of ACE inhibitors [18,19,20,21]. In particular, Pro407 lies in the hinge region between the two subdomains, where ligand-induced interactions can modulate active-site geometry and catalytic efficiency [22], while Phe570 provides an aromatic platform that frequently participates in π–π/π–alkyl contacts with high-affinity inhibitors [19,21]. Therefore, the observed interactions of gypenoside LI with these functionally important residues support its potential to interfere with ACE activity and reduce downstream angiotensin II–mediated signaling.

### 2.6. Three-Dimensional Quantitative Structure-Activity Relationship (3D-QSAR) Analysis

To further validate the predictive accuracy of the molecular docking results, a three-dimensional quantitative structure-activity relationship (3D-QSAR) study was conducted using 24 triterpenoids with known ACE inhibitory activities. All molecules were successfully aligned using the O3A algorithm with coherent aglycone scaffold superposition (Figure S1A). The CoMFA model with three principal components (PC = 3) achieved r^2^ = 0.943 on the training set and q^2^ = 0.582 (SDEP = 0.463) under leave-one-out cross-validation (Figure S1B). Y-scrambling validation with 100 permutations yielded a q^2^ distribution centered at −0.389 (maximum = 0.299), with 0 out of 100 permutations exceeding the observed q^2^ value (*p* < 0.01), confirming the statistical robustness of the model (Figure S1C). Using this validated model, gypenoside LI was predicted to have pIC_50_ = 4.98 (IC_50_ ≈ 10.5 μM). The CoMFA stdev×coeff contour maps revealed key structural features associated with ACE inhibitory activity (Figure S1D). In the steric field, green contours (favorable bulk regions) were predominantly located near the aglycone lateral face and central ring junction, while yellow contours (unfavorable bulk regions) flanked the sugar termini. In the electrostatic field, blue contours (regions where positive potential is favorable) aligned with glycosidic oxygen atoms, whereas red contours (regions where negative potential is favorable) appeared in opposing positions, suggesting a polarized binding environment. When gypenoside LI was overlaid onto these contour maps, its aglycone core occupied green steric regions while its hydroxyl-rich sugar chains were positioned near red electrostatic regions, consistent with the predicted moderate-to-good potency.

### 2.7. Molecular Dynamics (MD) Simulation Analysis

To assess the structural stability of the docked protein-ligand complexes over physiologically relevant timescales, 50 ns molecular dynamics simulations were performed for ACE-gypenoside LI and NOS2-ombuoside complexes. For the ACE-gypenoside LI complex, the protein backbone RMSD stabilized at 0.16–0.22 nm after initial equilibration, while the ligand RMSD plateaued at 0.25–0.30 nm without large fluctuations, indicating a stable binding mode (Figure S2A). RMSF analysis revealed that most residues exhibited low positional fluctuations (<0.15 nm), with elevated fluctuations observed only in flexible loop regions and the C-terminus (Figure S2B). The radius of gyration remained constant at 2.38–2.40 nm throughout the trajectory, confirming that the overall protein structure was preserved (Figure S2C). Similarly, the NOS2-ombuoside complex displayed stable protein backbone dynamics, although the ligand RMSD stabilized at a higher value of 0.7–0.9 nm, suggesting greater conformational flexibility within the binding pocket (Figure S3). MM-GBSA binding free energy calculations yielded ΔG_bind = −32.56 ± 6.84 kcal/mol for the ACE-gypenoside LI complex and ΔG_bind = −26.57 ± 4.90 kcal/mol for the NOS2-ombuoside complex, both indicating thermodynamically favorable binding (Figures S2 and S3). The ACE complex exhibited approximately 6 kcal/mol stronger binding affinity compared to the NOS2 complex, primarily attributable to more favorable van der Waals interactions (−61.69 vs. −41.47 kcal/mol) and non-polar solvation contributions (−9.20 vs. −4.97 kcal/mol). Binding free energy values converged to stable means after the equilibration phase in both systems.

## 3. Discussion

This study identified *G. pentaphyllum* as a potential multi-target agent for the treatment of COVID-19. Among the active compounds in *G. pentaphyllum*, ombuin and quercetin were highlighted for their strong associations with key inflammatory and cardiovascular targets. Five core targets—IL1B, IL6, TNF, ACE, and REN—were identified as central to *G. pentaphyllum*’s potential therapeutic action. These targets are critically involved in immune dysregulation and the renin–angiotensin system, both of which are central to COVID-19 pathophysiology [23,24]. Enrichment analyses confirmed that *G. pentaphyllum*’s predicted targets are involved in cytokine signaling and inflammatory pathways. Additionally, molecular docking supported the potential of ombuin to interact strongly with all five targets. Collectively, these findings suggest that *G. pentaphyllum* may exert therapeutic effects in COVID-19 through coordinated modulation of immune and cardiovascular pathways. Previous network pharmacology studies on *G. pentaphyllum* have primarily focused on metabolic, inflammatory, and oncological diseases [25,26,27,28,29,30,31,32,33,34,35,36,37,38,39,40,41,42,43,44,45,46,47,48,49,50,51,52,53,54,55,56,57,58,59], and, to date, coronavirus-related signaling or RAS-associated pathways have not been analyzed. In particular, our analysis newly identified targets such as NOS2, F2, and REN, which are mechanistically relevant to COVID-19 pathogenesis (Table 2).

Among the 19 intersecting targets identified between *G*. *pentaphyllum* compounds and COVID-19-associated genes, several key nodes—*IL1B, IL6, TNF, ACE,* and *REN*—emerged as central in both the protein–protein interaction and compound–target networks. These targets are well-recognized mediators in COVID-19 pathophysiology [60]. *IL1B, IL6,* and *TNF* are pro-inflammatory cytokines that contribute to the cytokine storm observed in severe COVID-19 cases, leading to acute respiratory distress and systemic inflammation [23]. On the other hand, *ACE* (angiotensin-converting enzyme) and *REN* (renin) are components of the renin–angiotensin system (RAS), which plays a dual role in both viral entry and vascular homeostasis [24,61]. SARS-CoV-2 is known to use *ACE2* as a receptor, and dysregulation of the RAS has been linked to increased disease severity [62]. The convergence of these two mechanistic axes—cytokine regulation and RAS modulation—supports the rationale for developing *G. pentaphyllum* as a multi-target therapeutic candidate for COVID-19.

An important feature of *G. pentaphyllum* is its distinctive phytochemical profile, comprising both flavonoids and triterpenoid saponins—two classes of compounds that are rarely co-enriched in a single medicinal plant [10]. In this study, flavonoids and triterpenoid saponins emerged as the representative active compounds based on network topology and docking affinity. These two compound types exhibited clearly differentiated target profiles and functional pathways. Flavonoids were primarily associated with immunological targets (e.g., *NOS2, TNF, IL2*), suggesting roles in anti-inflammatory and cytokine-modulating activities [63]. In contrast, triterpenoids interacted more strongly with targets related to the renin–angiotensin system (e.g., *ACE, AGTR2*), implicating its potential in regulating vascular homeostasis and interfering with viral entry processes [64]. This dual engagement of immune and cardiovascular axes supports a multi-faceted pharmacological action, whereby *G. pentaphyllum* may simultaneously modulate systemic inflammation and protect vascular integrity—two hallmarks of COVID-19 severity [65]. The presence of both compound types within a single extract positions *G. pentaphyllum* as a unique candidate for broad-spectrum antiviral intervention. However, the therapeutic relevance of these compounds is contingent upon their actual concentrations in *G. pentaphyllum* preparations and their bioavailability in vivo. A summary of available quantitative data on major phytochemical components and published pharmacokinetic profiles is provided in Supplementary Information to support further dose-feasibility assessment [66,67,68,69,70,71,72,73,74,75,76,77,78,79,80] **(**Tables S1 and S2). Additionally, the co-occurrence of flavonoids and triterpenoids in *G. pentaphyllum* may provide synergistic therapeutic benefits, as recent studies have demonstrated that these phytochemical classes can produce enhanced anti-inflammatory and antimicrobial effects when combined [64,81].

While this study focused on the acute phase of SARS-CoV-2 infection, the identified pharmacological actions of *G. pentaphyllum* may also have relevance for post-acute sequelae of COVID-19, often referred to as long COVID. Persistent inflammation, endothelial dysfunction, and renin–angiotensin system imbalance are key features shared between acute COVID-19 and long-term complications [2]. Persistent endothelial dysfunction and elevated inflammatory markers (IL-6, TNF-α) have been documented in long COVID patients for at least 6 months post-infection [82,83]. Importantly, natural polyphenol-rich compounds have shown clinical efficacy in reducing these inflammatory biomarkers and improving vascular function in post-COVID patients [84,85,86]. The dual-targeting capacity of *G. pentaphyllum*, via flavonoid-mediated cytokine regulation and triterpenoid-mediated vascular and hormonal modulation, offers a potentially valuable therapeutic strategy for addressing both acute symptoms and longer-term pathological changes. Additionally, the multi-target nature of *G. pentaphyllum* could prove beneficial in the face of emerging SARS-CoV-2 variants or related respiratory viral infections, where treatment approaches that act on host-based pathways rather than viral proteins may retain efficacy [87]. These findings support further preclinical validation and provide a rationale for considering *G. pentaphyllum* in broader antiviral and immunoregulatory contexts.

The reliability of our molecular docking predictions was further supported by complementary in silico validation approaches. 3D-QSAR analysis of 24 triterpenoid saponins demonstrated robust predictive performance (q^2^ = 0.58, r^2^ = 0.94), with gypenoside LI occupying sterically and electrostatically favorable regions around the ACE active site, consistent with strong inhibitory activity (Figure S1 and Table S4). Additionally, 100 ns MD simulations demonstrated stable protein-ligand interactions for both ACE–gypenoside LI and NOS2–ombuoside complexes, with minimal conformational drift (protein RMSD < 0.2 nm, ligand RMSD < 0.15 nm). MM-GBSA free energy calculations confirmed thermodynamically favorable binding throughout the trajectory, with ΔG_bind values of −32.6 ± 6.8 kcal/mol for ACE–gypenoside LI and −26.6 ± 4.9 kcal/mol for NOS2–ombuoside (Figures S2 and S3). These multi-layered computational validations strengthen confidence in the predicted interactions as plausible therapeutic targets worthy of experimental validation.

Despite the promising findings, this study has several limitations. First, all predictions were derived from in silico analyses, including network pharmacology and molecular docking, without experimental validation. Although these computational methods provide valuable insights into compound–target interactions and pathway associations, their predictions must be interpreted with caution until confirmed by in vitro and in vivo studies and supported by gene or protein expression analyses to verify coordinated modulation of immune and cardiovascular signaling. Second, the pharmacokinetics, bioavailability, and synergistic interactions of the active compounds within the complex *G. pentaphyllum* extract remain to be experimentally characterized, and the therapeutic feasibility in relation to effective dose ranges has not been evaluated. Moreover, variability in extraction solvents and plant parts across previous studies may influence phytochemical composition and affect translational relevance. Third, the study focused on molecular targets broadly associated with COVID-19 but did not distinguish between different clinical stages or viral variants, which may involve distinct pathological processes. Therefore, direct experimental evidence confirming the COVID-19-specific relevance of the identified targets is still needed. Future research should focus on experimental validation in relevant cellular and animal models, and assess the therapeutic efficacy of *G. pentaphyllum* in both acute and long-term manifestations of COVID-19 [88]. These efforts will be essential for translating computational predictions into clinically meaningful outcomes.

## 4. Materials and Methods

### 4.1. Screening for Active Compounds, Putative Target Proteins and COVID-19 Associated Genes

In this study, the active compounds of *G. pentaphyllum* Makino and their putative target proteins were retrieved from the KNApSAcK and PubChem databases [89]. To identify the chemical constituents of the herb, the keyword “*Gynostemma pentaphyllum*” was used in the KNApSAcK search interface, which serves as a unique repository encompassing a wide range of medicinal plants and their bioactive components. Putative target proteins for each active compound were predicted using the SwissTargetPrediction web tool (http://www.swisstargetprediction.ch/, accessed on 2 February 2024) [90], which utilizes the SMILES notation of chemical structures to estimate target affinity. Predicted targets with a probability score of zero were excluded from further analysis. Genes associated with COVID-19 were obtained from the MalaCards database (https://www.malacards.org/, accessed on 22 March 2024), a comprehensive disease-centric resource modeled after the GeneCards platform [91]. The keywords employed for gene retrieval included: “COVID-19,” “Severe COVID-19,” “Severe Acute Respiratory Syndrome,” “Critical COVID-19,” “Adult Respiratory Distress Syndrome,” and “Long COVID.”

### 4.2. Network Construction

The overlapping targets were identified by intersecting putative compound-related target proteins with COVID-19-associated genes. These common targets were considered potential therapeutic targets for COVID-19. The compound–target interaction network was visualized using Cytoscape (version 3.10.1) [92], and key bioactive compounds were identified based on network topology parameters, such as degree, betweenness, and closeness centrality. Additionally, a protein–protein interaction (PPI) network of the common targets was identified using the STRING web tool (https://string-db.org/, accessed on 29 March 2024) and filtered with an interaction score of less than 0.5 [92]. Targets were prioritized based on their degree values, which indicate their relative importance within the network.

### 4.3. Gene Ontology (GO) Analysis and Kyoto Encyclopedia of Genes and Genomes (KEGG) Pathway Enrichment Analysis

To further elucidate the pathogenesis of COVID-19 and clarify the underlying mechanism of action of *G. pentaphyllum*, the intersecting genes were subjected to functional enrichment analysis using ShinyGO web tool (https://bioinformatics.sdstate.edu/go77/, accessed on 30 March 2024) [93]. This analysis included Gene Ontology (GO) enrichment, which encompasses biological processes, cellular components, and molecular functions, as well as Kyoto Encyclopedia of Genes and Genomes (KEGG) pathway enrichment.

### 4.4. Molecular Docking

Molecular docking studies were performed to evaluate the binding affinity and interaction patterns between the bioactive compounds of *G. pentaphyllum* and their key protein targets associated with COVID-19. SDF files of the compounds were obtained from the PubChem database and converted to PDB files using PyMOL, and subsequently to PDBQT files using AutoDockTools 1.5.7. The grid box size was defined as 60 × 60 × 60 Å for all targets with a grid spacing of 0.375 Å. The x, y, and z coordinates for each protein are summarized in Table S5. It was run at a ‘num_modes’ of 10 and an ‘energy_range’ of 4. Energy minimization was performed using ChemBio3D Ultra 12.0 with the MMFF94 force field until the root-mean-square (RMS) gradient reached a value of 0.01 kcal/mol·Å. The compounds without 3D structures were converted using Avogadro2 (version 1.102.1) [94]. The three-dimensional (3D) structure of the major proteins was retrieved from the Protein Data Bank (PDB) with the following PDB IDs: ACE (1O86), AGTR2 (5UNG), ALOX5 (3V99), EGFR (2ITX), F2 (1PPB), IL1β (8C3U), IL2 (1M48), NOS2 (2NSI), PLA2G2A (3U8I), PLG (1CEB), TNF (2AZ5), VDR (1DB1), Mpro (6LU7), RdRp (6M71), and Spike protein (6WPT). Selected proteins had water molecules removed, hydrogen atoms added, and Kollman charges assigned using AutoDockTools [95]. Molecular docking was performed using AutoDock Vina, and the results were visualized using Discovery Studio Client [95]. The docking protocol was validated by re-docking the co-crystallized ligands into their native binding sites, and comparing the resulting poses with the corresponding experimental structures using RMSD analysis calculated with Discovery Studio Client.

### 4.5. Three-Dimensional Quantitative Structure-Activity Relationship (3D-QSAR)

Twenty-four triterpenoids with known ACE inhibitory activity were collected from literature sources [96], and their IC_50_ values were converted to pIC_50_ (−log_10_ IC_50_). Three-dimensional structures were generated, energy-minimized using the MMFF94 force field, and aligned to Protopanaxatriol using RDKit’s O3A algorithm. Comparative Molecular Field Analysis (CoMFA) was performed using Open3DQSAR v2.3 with a 2.0 Å grid (23 × 18 × 16 nodes) to calculate steric (Lennard-Jones) and electrostatic (Gasteiger charges) fields. After field processing (cutoff ± 30, zero level 0.05, sdcut 2.0) and Block Unscaled Weighting, partial least squares (PLS) regression was performed with three principal components (PC = 3) determined by leave-one-out cross-validation (LOO-CV). Model robustness was validated by Y-scrambling (100 permutations). Standard-deviation × coefficient contour maps were visualized in PyMOL 3.1.6 with green/yellow (steric favorable/unfavorable) and blue/red (electrostatic positive/negative favorable) isosurfaces.

### 4.6. Molecular Dynamics (MD) Simulation

Molecular dynamics simulations were performed using GROMACS 2025.3 with CUDA support. Two protein-ligand complexes (ACE-Gypenoside LI and NOS2-Ombuoside) were prepared with complete protein coordinates, cofactors (heme and tetrahydrobiopterin for NOS2), and explicit hydrogens. Systems were parameterized using a biomolecular force field, solvated in periodic cubic boxes with explicit water, and neutralized with counterions. After energy minimization (steepest descent with positional restraints) and equilibration (NVT pre-equilibration and restrained thermalization/pressure equilibration), production simulations were conducted for 50 ns using the Verlet cutoff scheme, PME for long-range electrostatics, LINCS constraints for hydrogen bonds, and a 2-fs time step. Binding free energies were estimated using MM-GBSA (gmx_MMPBSA, igb = 5, gbsa = 2) on 200 snapshots from the final 50 ns, calculating gas-phase van der Waals (ΔE_vdW) and electrostatic (ΔE_elec) interactions, as well as polar (ΔG_polar) and non-polar (ΔG_non-polar) solvation contributions.

## 5. Conclusions

This study demonstrated the potential of *G. pentaphyllum* extract as a complementary therapeutic candidate for COVID-19 through an integrative in silico approach. Through network pharmacology and molecular docking, 59 curated compounds and 19 COVID-19–overlapping targets were identified. Furthermore, we found six flavonoids and triterpenoid saponins as key active compounds, each exhibiting distinct target profiles—flavonoids being primarily linked to immunological and inflammatory pathways (*IL1B*, *IL6*, *TNF*, *NOS2*), and triterpenoids to the renin–angiotensin system and host entry regulation (*ACE*, *REN*, *AGTR2*). These dual mechanistic pathways reflect the multi-target nature of the extract and its relevance to COVID-19 pathogenesis. To translate these findings, future studies should validate target engagement experimentally and assess whether representative flavonoid and saponin fractions show complementary or synergistic effects. Establishing exposure–activity relationships through quantitative profiling and pharmacokinetic evaluation will also be essential. Overall, the findings in this study provide a preliminary mechanistic basis that supports a hypothesis for further experimental validation of *G. pentaphyllum* as a multi-target herbal candidate with potential relevance to both acute and persistent manifestations of COVID-19 by modulating immuno-inflammatory signaling and RAS/host-entry–related proteins.

## Figures and Tables

**Table 1:** Docking affinities between major compounds and targets associated with active compounds of *G. pentaphyllum* and COVID-19. All docking simulations were performed in duplicate (n = 2), and RMSD values between runs were calculated.

		Targets (kcal/mol) [RMSD] (Å)														
Type	Compounds (Code)	ACE [0.730]	AGTR2 [0.127]	ALOX5 [1.902]	EGFR [0.925]	F2 [0.919]	IL1B [0.885]	IL2 [2.137]	NOS2 [1.377]	PLA2G2A [N.C.]	PLG [0.599]	TNF [2.298]	VDR [0.790]	Mpro [2.173]	RdRp [0.340]	Spike Protein [0.356]
Triterpenoid	Damulin B (GPM24)	−10.3 ± 0.0	−9.5 ± 0.5	−9.2 ± 0.0	−8.2 ± 0.2	−7.9 ± 0.1	−6.5 ± 0.1	−6.5 ± 0.5	−8.8 ± 0.1	−6.5 ± 0.2	−6.1 ± 0.2	−8.1 ± 0.0	−7.6 ± 0.0	−7.3 ± 0.0	−11.4 ± 0.0	−6.8 ± 0.6
	Gypenoside LI (GPM32)	−10.2 ± 0.0	−9.5 ± 0.0	−9.5 ± 0.0	−8.1 ± 0.0	−7.7 ± 0.1	−6.4 ± 0.0	−6.5 ± 0.0	−9.5 ± 0.0	−6.8 ± 0.1	−6.4 ± 0.1	−7.7 ± 0.1	−7.3 ± 0.1	−7.4 ± 0.1	−10.7 ± 0.0	−6.9 ± 0.4
	Gypensapogenin E (GPM30)	−10.0 ± 0.0	−9.9 ± 0.0	−10.6 ± 0.8	−8.3 ± 0.0	−7.8 ± 0.0	−6.6 ± 0.0	−7.5 ± 0.3	−8.7 ± 0.1	−7.0 ± 0.0	−6.6 ± 0.2	−8.7 ± 0.3	−7.6 ± 0.3	−8.0 ± 0.4	−9.5 ± 0.1	−7.2 ± 0.1
Flavonoid	Ombuin (GPM10)	−8.5 ± 0.0	−7.9 ± 0.1	−8.7 ± 0.0	−8.0 ± 0.0	−7.3 ± 0.0	−8.2 ± 0.0	−6.1 ± 0.0	−8.2 ± 0.0	−6.4 ± 0.0	−6.3 ± 0.0	−7.5 ± 0.0	−8.6 ± 0.0	−7.0 ± 0.0	−8.4 ± 0.0	−6.7 ± 0.1
	Ombuoside (GPM15)	−10.1 ± 0.0	−9.3 ± 0.1	−9.5 ± 0.0	−8.1 ± 0.0	−8.6 ± 0.0	−6.5 ± 0.0	−6.4 ± 0.4	−9.7 ± 0.0	−6.5 ± 0.3	−7.2 ± 0.1	−8.2 ± 0.0	−7.5 ± 0.0	−7.7 ± 0	−10.4 ± 0.0	−7.3 ± 0.1
	Rutin (GPM23)	−9.8 ± 0.0	−10.0 ± 0.0	−9.3 ± 0.1	−9.2 ± 0.0	−9.0 ± 0	−7.8 ± 0.0	−6.8 ± 0.0	−9.8 ± 0.0	−7.2 ± 0.0	−6.5 ± 0.5	−7.8 ± 0.3	−7.9 ± 0.1	−7.7 ± 0.1	−10.7 ± 0.0	−7.7 ± 0.0
Reference	Lisinopril (ACE inhibitor)	−7.7 ± 0.1	−7.1 ± 0.2	−7.2 ± 0.1	−6.4 ± 0.0	−6.7 ± 0.1	−6.3 ± 0.0	−5.5 ± 0.0	−7.8 ± 0.3	−5.8 ± 0.1	−5.1 ± 0.4	−7.4 ± 0.1	−6.8 ± 1.0	−6.0 ± 0.1	−7.6 ± 0.3	−5.9 ± 0.1
	Remdesivir (RdRp inhibitor)	−6.0 ± 0.0	−5.2 ± 0.0	−6.2 ± 0.1	−5.1 ± 0.0	−5.6 ± 0.3	−5.4 ± 0.1	−4.7 ± 0.2	−6.3 ± 0.1	−4.8 ± 0.0	−5.1 ± 0.1	−5.1 ± 0.0	−6.2 ± 0.2	−5.7 ± 0.0	−6.7 ± 0.0	−5.1 ± 0.1

**Table 2:** KEGG pathways of diseases associated with *G. pentaphyllum* analyzed through network pharmacology.

Category	Disease	Pathways	Key Target	Refs.
Liver	NAFLD and NASH	Apoptosis; Non-alcoholic fatty liver disease; PI3K-Akt signaling pathway; Lipid and atherosclerosis	AKT1, GSK3B	[25]
	Hepatic fibrosis	Proteoglycans in cancer; PI3K–Akt signaling pathway; EGFR tyrosine kinase inhibitor resistance; EGFR signaling pathway	AKT1, IL6, STAT3, EGFR	[26,27]
	Liver injury	Apoptosis signaling pathway	STAT3, HIF1A, PTGS2, EGFR, PPARG, MTOR, AKT1, ESR1, TNF	[28]
	Metabolism-associated fatty liver disease	NF-κB signaling pathway; PI3K-Akt signaling pathway; TNF signaling pathway; HIF-1 signaling pathway	TNF, IL6, PTGS2, TP53, CCL2, VEGFA	[29]
	Non-alcoholic fatty liver disease	HIF-1 signaling pathway; AGE-RAGE signaling pathway in diabetic complications; Chagas disease; Beta-oxidation pathway	IL6, PTGS2, NFKB1, CDK6, SERPINE1, ADRB2, PCNA, TOP2A, TYMS, PPARA	[30,31]
Heart	Heart failure	PI3K/Akt signaling pathway; MAPK signaling pathway; Ras signaling pathway	MAPK, EGFR, PI3KCA, MCL1	[32]
Lung	Non-small-cell lung cancer	Chemical carcinogenesis receptor activation; Lipid and atherosclerosis; Human cytomegalovirus infection; Pyrimidine metabolism; Pantothenate and CoA biosynthesis	MMP9, STAT3, TYMS, MYC, ESR1, HIF1A	[33,34]
	Acute lung injury	TNF-α signaling pathway; NF-κB signaling pathway	CXCL2, CXCL1, CXCL10, CCL2, TNFAIP3, SOCS3, IFIT2, TLR2, IL1, IL6, IRF7,	[35]
	Lung cancer	MAPK14/STAT3 signaling pathway	STAT3, MAPK14, EGFR, TYMS	[36]
Brain/nervous system	Alzheimer’s disease	Amoebiasis; African trypanosomiasis; Cytokine-cytokine receptor interaction	EGFR, IL1B, IL6, NOS3, PON1	[37]
	Depression	HIF-1 signaling pathway; PI3K-Akt signaling pathway; TNF signaling pathway	SIRT1	[38]
	Glioma	EGFR tyrosine kinase inhibitor resistance; Prolactin signaling pathway; VEGF signaling pathway	STAT3, HSP90AA1, IL6, ESR1, PIK3CA, MTOR, PTPN11, MAPK3, BCL2L1, PTGS2	[39]
Cancer	Breast cancer	PI3K-Akt signaling pathway; Breast cancer pathway; EGFR tyrosine kinase inhibitor resistance	ALB, EGFR, ESR1, AR, PGR, HSP90AA1	[40]
	Osteosarcoma	PI3K-Akt signaling pathway; MAPK signaling pathway; Cellular senescence	CCND1, RELA, TNF	[41]
	Esophageal cancer	PI3K-Akt signaling pathway; MAPK signaling pathway; IL-17 signaling pathway	AKT1, TP53, VEGFA	[42]
	Cervical intraepithelial neoplasia	Cancer; Fluid shear stress and atherosclerosis; Lipid and atherosclerosis	IL6, IL1B, TNF, TP53, PTGS2	[43]
	Renal cell carcinoma	Cancer; Prolactin signaling pathway; PI3K-Akt signaling pathway, Ras/MAPK pathways	VEGFA, PIK3CA, JAK2, CCND1, MAPK3, EGFR, CASP3, HRAS, STAT3, SRSC	[44,45]
	Anaplastic thyroid cancer	Cancer; PI3K-Akt signaling pathway; Proteoglycans in cancer	HSP90AA1, SRC, CASP3	[46]
	Bladder cancer	PI3K-Akt signaling pathway; Pathway in cancer; Proteoglycans in cancer	VEGFA, STAT3, PI3KCA	[47]
	Gastric cancer	Glycerophospholipid metabolism; Alanine, aspartate and glutamate metabolism; Central carbon metabolism in cancer; PD-L1 checkpoint in cancer; T-cell receptor signaling pathway; Th17 differentiation	ALB, EGFR, HSP90AA1, MMP9, ESR1, IGF1, PPARG, ANXA5	[48,49]
	Ophthalmopathy	JAK–STAT signaling pathway	STAT1, STAT3, STAT4	[50]
	Retinitis Pigmentosa	Cancer; HIF-1 signaling pathway; TNF signaling pathway; PI3K-Akt signaling pathway	FGF2, IL6, MAPK14, MMP2, MMP9, SLC2A1	[51]
	Pancreatic cancer	Cancer; AGE-RAGE signaling pathway in diabetic complications; hepatitis C; IL-17 signaling pathway; Platinum drug resistance	MET, SPP1, ADRA1A, HMOX1, CCL2, ICAM1, VCAM1, BCL2, PRKCB, NCF1,	[52]
Metabolic/Endocrine	Type II Diabetes	Insulin resistance; PI3K-Akt signaling pathway; FOXO signaling pathway	STAT3, PIK3CA, AKT1, EGFR, VEGFA, INSR	[53]
	Hyperlipidemia	Lipid metabolism and atherosclerosis; TNF signaling pathway; PI3K-Akt signaling pathway	NCOA2, NR3C2, PGR, PPARG, IL6, PPARG, VEGFA	[54]
	Diabetic cataracts	AGE-RAGE signaling pathway in diabetic complications; Fluid shear stress and atherosclerosis; Pathways in cancer	IL6, IL1B, TNF, CASP3, MMP9, AKT1, TGFB1, HIF1A, PTGS2, JUN	[55]
	Glucose metabolic disorders	AGE-RAGE signaling pathway	SRC, PIK3CA, HRAS, AKT1	[56]
	Obesity	AGE-RAGE signaling pathway; glycerolipid metabolism; lipid and atherosclerosis metabolism; Cholesterol metabolism	HMGCR, ACE, LIPC, LIPG, PPARA, PPARD, PPARG	[57]
Immune/Inflammatory	Acute Pharyngitis	Cancer; PI3K-Akt signaling pathway; JAK–STAT signaling pathway	EGFR, STAT3, MAPK3, SRC, AKT1	[58]
	Diarrhea	Cancer; PI3K-Akt pathway; MAPK pathway	AKT1, STAT3, SRC, ESR1, EGFR, HSP90AA1	[59]
Respiratory system	COVID-19	Coronavirus disease; renin–angiotensin system	NOS2, F2, REN *, IL1B, IL6, TNF, ACE, EGFR	This study

* Underlined targets indicate those newly identified in the present study.

## Data Availability

The original contributions presented in this study are included in the article/Supplementary Material. Further inquiries can be directed to the corresponding authors.

## Supplementary Materials

The following supporting information can be downloaded at: https://www.mdpi.com/article/10.3390/ph18121851/s1, Figure S1. Validation of 3D-QSAR CoMFA model and contour analysis for ACE inhibitors. Figure S2. Molecular dynamics simulation validation of ACE-Gypenoside LI complexes. Figure S3. Molecular dynamics simulation validation of NOS2-Ombuoside complexes. Table S1. Content of components determined in *G. pentaphyllum* from literature. Table S2. Pharmacokinetic parameters of *G. pentaphyllum* extracts and major components following oral administration in animal models. Table S3. The oral bioavailability (OB) and drug-likeness (DL) of major components from *G. pentaphyllum* extracts. Table S4. Statistical parameters and computational settings for 3D-QSAR and molecular dynamics validation. Table S5. The grid box centers of target proteins used in molecular docking simulations.
