# Supplementary material for: Therapeutic Potential of Gynostemma pentaphyllum (Thunb.) Makino Against COVID-19 Identified Through Network Pharmacology
Source: Pharmaceuticals (Basel). 2025 Dec 4;18(12):1851. doi: 10.3390/ph18121851 (PMC12735714; doi:10.3390/ph18121851)
Supplement: Supplementary file 1 [file pharmaceuticals-18-01851-s001.zip › pharmaceuticals-3911347-supplementary.pdf]

Therapeutic Potential of *Gynostemma pentaphyllum* (Thunb.) Makino Against COVID-19 Identified Through Network Pharmacology

Min Ho Kim [1,2,†], Jin Ah Won [1,†], Jun Sang Yu[1,3] , Su Min Kim [1], Dong Keun Lee [4], Xiang-Lan Piao [5,*] and Hye Hyun Yoo [1,*]

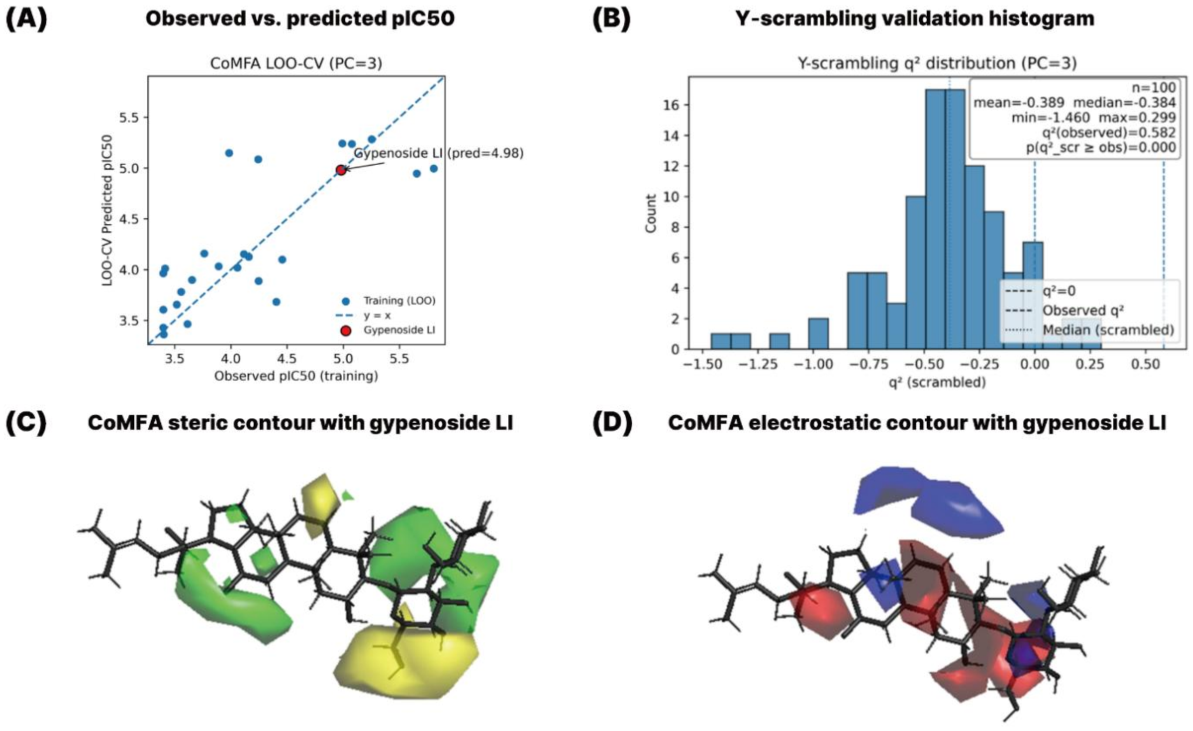

Figure S1. Validation of 3D-QSAR CoMFA model and contour analysis for ACE inhibitors. (**A**) Correlation between observed and LOO-predicted pIC_{50} values for the training set of 24 triterpenoid saponins. The solid line represents the ideal correlation (slope = 1), demonstrating robust model performance (q^2 = 0.58, r^2 = 0.94). (**B**) Distribution of q^2 values from 100 Y-scrambling permutations, confirming the statistical robustness of the CoMFA model. The vertical dashed line indicates the original model q^2 (0.58), which lies well outside the randomized distribution. (**C**) CoMFA steric contour map superimposed on gypenoside LI bound to ACE. Green regions indicate areas where increased steric bulk enhances ACE inhibitory activity, while yellow regions indicate unfavorable steric interactions. (**D**) CoMFA electrostatic contour map with gypenoside LI. Blue regions represent areas where positive charge is favorable, and red regions indicate preference for negative charge. The contour maps demonstrate that gypenoside LI occupies structurally favorable regions consistent with strong ACE inhibition.

(A) RMSD of ACE (protein) (B) RMSD of gypenoside LI (ligand)

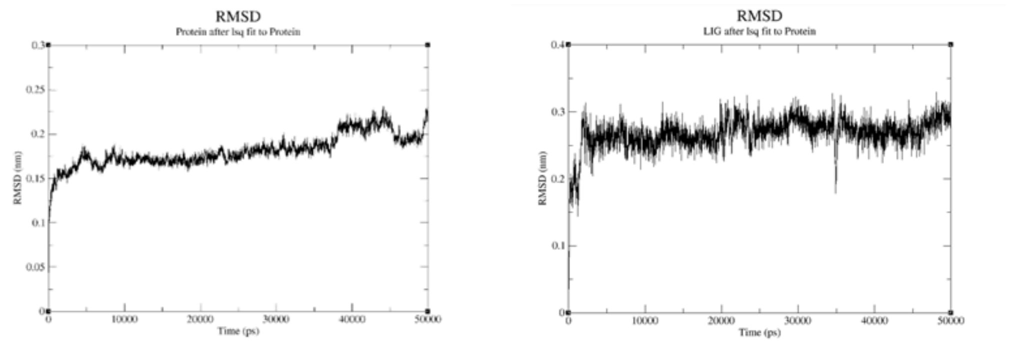

(C) MM-GBSA binding energy evolution of ACE

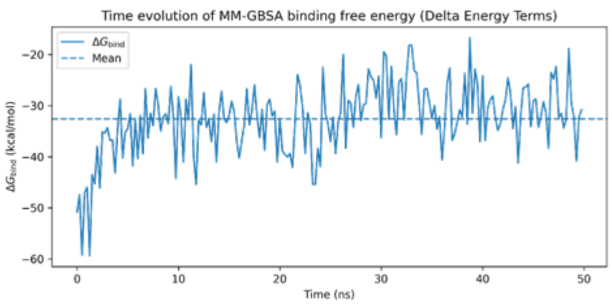

Energy decomposition table

Parameter	Mean ± SD (kcal/mol)
ΔE_vdw	−61.7 ± 4.2
ΔE_elec	−37.6 ± 11.3
ΔG_polar	75.9 ± 9.0
ΔG_npol	−9.2 ± 0.5
ΔG_bind	−32.6 ± 6.8

Figure S2. Molecular dynamics simulation validation of ACE-gypenoside LI complexes. (**A**) Time evolution of backbone RMSD for the ACE protein during 100 ns MD simulation with gypenoside LI. RMSD stabilizes below 0.2 nm after initial equilibration (~10 ns), indicating structural stability of the protein-ligand complex. (**B**) Ligand RMSD of gypenoside LI relative to the ACE binding site throughout the simulation. The ligand remains stable with RMSD < 0.15 nm, demonstrating minimal conformational drift. (**C**) Time evolution of MM-GBSA binding free energy (ΔG_bind) for the ACE–gypenoside LI complex. Energy values remain stable around a favorable mean after equilibration, supporting thermodynamic favorability of the interaction.

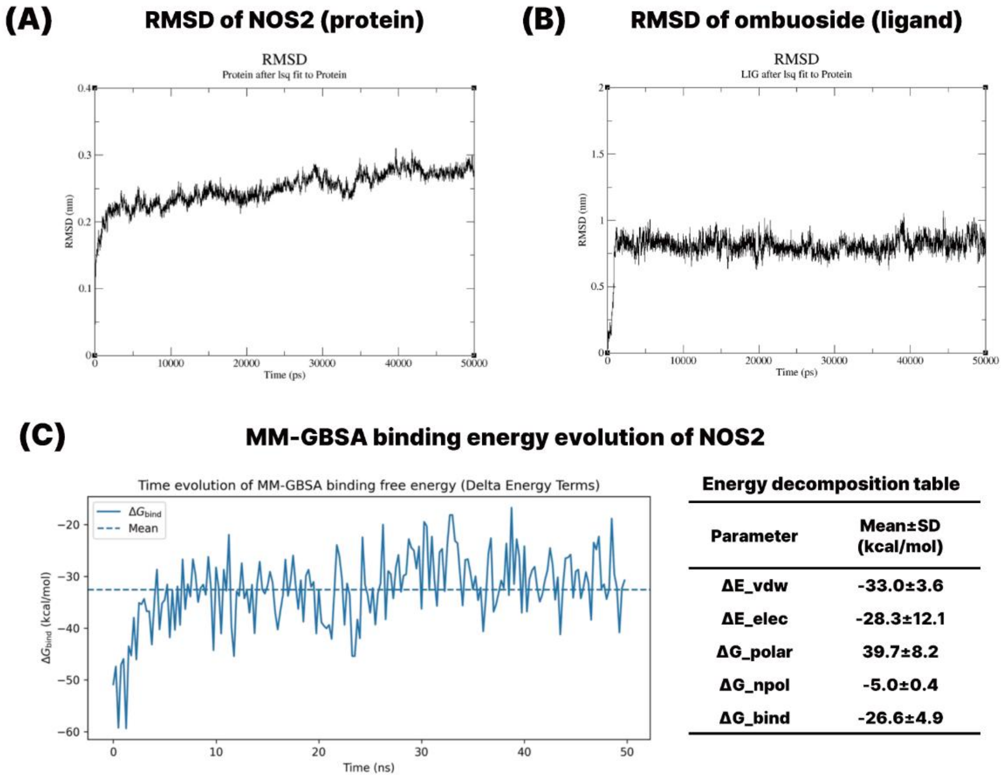

Figure S3. Molecular dynamics simulation validation of NOS2-ombuoside complexes. (**A**) Backbone RMSD of NOS2 protein complexed with ombuoside over 100 ns. The protein structure equilibrates rapidly and maintains stability throughout the trajectory. (**B**) Ligand RMSD of ombuoside in the NOS2 binding pocket, showing consistent binding mode throughout the simulation. (**C**) MM-GBSA binding free energy trajectory for the NOS2–ombuoside complex, demonstrating sustained favorable binding energy throughout 100 ns simulation. All analyses confirm stable and energetically favorable protein-ligand interactions predicted by molecular docking.

Table S1. Content of components determined in *G. pentaphyllum* from literature.

Plant Part (Form)	Extraction Solvent	Component	Content (mg/g)	Ref.
Leaves	80% methanol	Ginsenoside Rb3	2.25	[66]
		Ginsenoside Rd	0.92	
		Ginsenoside Rk1	0.08	
		Ginsenoside Rg5	0.01	
Leaves	80% methanol	Gypenoside L	0.05	[67]
		Gypenoside LI	0.02	
		Damulin B	0.03	
		Damulin A	0.21	
Leaves	80% ethanol	Gypenoside LI	17.89	[68]
		Gypenoside LVI	11.46	
		Gypenoside L	5.98	
		Gypenoside XLVI	5.14	
		Damulin B	4.94	
		Ginsenoside Rd	3.87	
		Damulin A	3.27	
		20(S)-ginsenoside Rg3	0.17	
Leaves	70% ethanol	Rutin	12.91	[69]
		Ombuoside	0.54	
		Isorhamnetin-3-O-β-D-Rutinoside	0.45	
		4'-O-methyl-kaempferol-3-O-Rutinoside	0.34	
		Quercetin-3-O-β-D-glucoside	0.06	
		Kaempferol-3-β-D-O-rutinoside	0.04	
Leaves	30% ethanol	Ginsenoside III	52.92	[70]
		Rutin	23.11	
Not specified (Dried Herb)	95% ethanol	Gypenoside XVII	9.21	[71]
		Isoquercitrin	4.30	
		Linoleic acid	3.62	
		Gypenoside A	2.75	
		Gypenoside LXXIV	2.33	
		Kaempferol	2.07	
		Narcissoside	1.78	

		Quercetin	1.62	
		Isorhamnetin	0.57	
Not specified (Powder)	Methanol	Gypenoside IV<br>Kaempferol-rhamno-hexoside<br>Gypenoside VIII<br>Quercetin-rhamno-hexoside<br>Gypenoside XLIV<br>Quercetin-di-(rhamno)-Hexoside<br>Rutin	4.53<br>4.45<br>3.37<br>1.80<br>1.66<br>1.25<br>0.17	[72]
Not specified (Powder)	Hexane–Acetone–Ethanol–Toluene (10:7:6:7, v/v/v/v)	Pheophytin a	2.51	[73]
		Pheophytin b	0.32	
		Chlorophyll b	0.29	
		Chlorophyll a	0.11	
		Pheophytin a′	0.11	
		Hydroxypheophytin a	0.09	
		Pyropheophytin a	0.08	
		Hydroxypheophytin a′	0.07	
		Hydroxychlorophyll a	0.02	
		Hydroxychlorophyll b	0.02	
		Pheophytin b′	0.01	
		Hydroxypheophytin b	0.01	
		Chlorophyll a′	0.01	
		Chlorophyll b′	0.01	
		Hydroxypheophytin b′	0.01	

Data are presented as mean values.

Table S2. Pharmacokinetic parameters of *G. pentaphyllum* extracts and major components following oral administration in animal models.

Type	Drug	Animal	Dose	Component	AUC_{0-t} (ng·h/mL)	BA	Ref.
Extracts	Total extract	Rats (SD, Male)	875 mg/kg (P.O.)	Narcissoside	122.4*	-	[71]
				Linoleic acid	326.0*		
				Gypenoside A	476.1*		
				Gypenoside XVII	476.1*		
				Gypenoside LXXIV	341.5*		
	Saponin extract	Rats (SD, Male)	120 mg/kg (P.O.)	Gypenoside LXIX	1114.9 ± 800.0	-	[74]
				Gypenoside CXVI	1377.7 ± 876.7		
				Gypenoside LVI	475.7 ± 281.4		
				Gypenoside XLIII	694.0 ± 519.4		
				Ginsenoside Rb2	1993.3 ± 1193.7		
				Gypenoside XLVI	762.1 ± 623.2		
				Ginsenoside Rb3	1427.7 ± 877.8		
				Gypenoside LXXXVII	1433.2 ± 1074.2		
				Gypenoside CVII	1127.5 ± 734.8		
				Gypenoside XV	868.2 ± 597.6		
				Ginsenoside Rd	942.4 ± 609.0		
				Gypenoside XCI	243.9 ± 171.8		
				Gypenoside LVII	182.4 ± 82.9		
	Saponin extract	Rats (SD, Male)	136 mg/kg (P.O.)	Gypenoside LVI	15789.0 ± 10409.0	-	[75]
				Gypenoside XLVI	11917.0 ± 6591.0		
Compounds	2α-OH-protopanaxadiol	Rats (SD, Male)	50 mg/kg (P.O.)	2α-OH-protopanaxadiol	3827.0 ± 806.0	-	[75]
	Gypenoside XLIX	Rats (SD, Male)	1 mg/kg (I.V.)	Gypenoside XLIX	2895.0 ± 576.0	-	[76]

		2 mg/kg (I.V.)		6777.0 ± 1478.0		
		4 mg/kg (I.V.)		11229.0 ± 1086.0		
Gypenoside A<br>Gypenoside XLIX	Rats (SD, Male)	5 mg/kg (P.O.)<br>1 mg/kg (I.V.)<br>5 mg/kg (P.O.)<br>1 mg/kg (I.V.)	Gypenoside A<br>Gypenoside XLIX	14.9 ± 2.4<br>332.9 ± 31.2<br>13.7 ± 2.5<br>1923.5 ± 62.5	0.90%<br>0.14%	[77]
Gypenoside XLVI	Rats (SD, Male)	10 mg/kg (P.O.)<br>1 mg/kg (I.V.)	Gypenoside XLVI	1032.8 ± 334.8<br>2213.9 ± 561.5	4.56%	[78]
Gypenoside TN-1	Mice (C57BL/6, Male)	25 mg/kg (P.O.)<br>1 mg/kg (I.V.)	Gypenoside TN-1	1061.7 ± 338.3<br>573.4 ± 84.2	10.28%	[79]
Gypenoside XLIX	Mice (C57BL/6, Male)	50 mg/kg (P.O.)<br>5 mg/kg (I.V.)	Gypenoside XLIX	400.0 ± 86.7<br>5250.1 ± 1108.7	1.40%	[80]

Data are presented as mean ± SD. AUC, area under the plasma concentration–time-curve; BA, Bioavailability; P.O, oral administration; I.V., Intravenous administration. [a]AUC value was corrected based on the plasma concentration data due to an incorrect unit reported in the original article.

Table S3. The oral bioavailability (OB) and drug-likeness (DL) of major components from *G. pentaphyllum* extracts.

Component	Oral Bioavailability (OB)	Drug-Likeness (DL)
Ombuin	0.55	Yes (Lipinski, Ghose, Veber, Egan, Muegge)
Gypensapogenin E	0.55	Yes (Lipinski, Veber)
Damulin B	0.17	No
Ombuoside	0.17	No
Rutin	0.17	No
Gypenoside LI	0.17	No

Table S4. Statistical parameters and computational settings for 3D-QSAR and molecular dynamics validation.

Model	Parameter	Value
3D-QSAR (CoMFA)	Training set size	24 compounds (pIC_{50}: 3.398–5.804)
	LOO cross-validation (q^2)	0.5818
	Model fit (r^2)	0.9429
	Standard error (SDEP)	0.463
	PLS components	3
MD Simulation	Software	GROMACS 2025.3 + CUDA 12.4
	Force field	AMBER/CHARMM
	Water model	TIP3P
	Simulation time	100 ns (each complex)
	Temperature	310 K
	Pressure	1 bar

Summary of key statistical metrics for the 3D-QSAR CoMFA model, including training set composition, cross-validation statistics (q^2, r^2), and model error (SDEP). Computational parameters for molecular dynamics simulations are also provided, including software version, force field, solvation model, simulation duration, and thermodynamic conditions used for trajectory analysis of ACE–gypenoside LI and NOS2–ombuoside complexes.

Table S5. The grid box centers of target proteins used in molecular docking simulations.

Target Protein	x	y	z
ACE	40.543	37.234	43.570
AGTR2	11.648	8.390	-17.754
ALOX5	17.839	-77.459	-30.057
EGFR	-51.672	-3.786	-26.993
F2	2.756	22.710	21.521
IL1B	-16.330	-9.291	-47.348
IL2	9.546	9.206	-1.933
NOS2	11.714	61.209	24.944
PLA2G2A	30.798	15.086	11.515
PLG	18.186	-1.730	32.476
TNF	-18.626	74.438	35.945
VDR	13.314	20.649	42.064
Mpro	-13.293	12.584	64.788
RdRp	94.102	84.899	100.976
Spike protein	-32.284	26.199	5.569
